# SIMMAP: Stochastic character mapping of discrete traits on phylogenies

**DOI:** 10.1186/1471-2105-7-88

**Published:** 2006-02-23

**Authors:** Jonathan P Bollback

**Affiliations:** 1 Bioinformatics Center, University of Copenhagen, Universitetsparken 15, Building 10, 2100 Copenhagen Ø, Denmark

## Abstract

**Background:**

Character mapping on phylogenies has played an important, if not critical role, in our understanding of molecular, morphological, and behavioral evolution. Until very recently we have relied on parsimony to infer character changes. Parsimony has a number of serious limitations that are drawbacks to our understanding. Recent statistical methods have been developed that free us from these limitations enabling us to overcome the problems of parsimony by accommodating uncertainty in evolutionary time, ancestral states, and the phylogeny.

**Results:**

SIMMAP has been developed to implement stochastic character mapping that is useful to both molecular evolutionists, systematists, and bioinformaticians. Researchers can address questions about positive selection, patterns of amino acid substitution, character association, and patterns of morphological evolution.

**Conclusion:**

Stochastic character mapping, as implemented in the SIMMAP software, enables users to address questions that require mapping characters onto phylogenies using a probabilistic approach that does not rely on parsimony. Analyses can be performed using a fully Bayesian approach that is not reliant on considering a single topology, set of substitution model parameters, or reconstruction of ancestral states. Uncertainty in these quantities is accommodated by using MCMC samples from their respective posterior distributions.

## Background

Mapping characters on phylogenies provides a historical framework essential to our understanding of the evolution of behavioral, morphological, ecological, and molecular traits [1-3]. In addition, knowledge of the patterns of character change along a phylogeny has proven valuable to our understanding of the process of selection [4,5] and neutral evolution [6-8].

The parsimony method is almost universally used to map characters. Indeed, user friendly programs that implement the parsimony method are available (e.g., MacClade, [9] and Mesquite, [10]). Parsimony attempts to minimize the number of changes along a phylogeny in a manner that is consistent with the character states observed at the tips. This is accomplished by identifying character states at the internal nodes of the tree that produce the minimum number of changes. Unfortunately, parsimony has a number of significant limitations. First, and most importantly, it assumes that along any branch that only a single change has occurred, regardless of the evolutionary time spanned. Second, parsimony can severely underestimate the variance in ancestral state assignments by considering only those ancestral reconstructions that minimize the number of changes on the phylogeny. Finally, parsimony does not provide a framework for accommodating uncertainty in the phylogenetic reconstruction.

Two alternative approaches have attempted to address some of the drawbacks inherent to parsimony: maximum likelihood [2] and Bayesian inference [11]. Both use stochastic models of character state change and thus can explicitly accommodate uncertainty in ancestral states. The Bayesian approach accommodates phylogenetic uncertainty by evaluating character maps on trees sampled from the posterior distribution [11,12]. However, these methods still rely on parsimony to determine the identity of the character state changes and thus suffer from the restriction of placing, at most, a single state change along a branch. One way of avoiding these problems is to estimate the rates of character change using maximum likelihood rather than explicitly mapping changes to the tree [13]. While, for many questions, this approach will be appropriate, it unfortunately lacks information about the location and identity of specific character state changes on the phylogeny.

### Bayesian mutational mapping

The mutational mapping approach [14-16], recently introduced, does not suffer from the problems of parsimony or the limitations of hybrid methods. In addition, this approach estimates the timing (placement) of changes along branches, providing a novel level of inference previously unavailable. While this Bayesian approach offers a better framework for addressing questions about character evolution we should recognize that as with all methods factors such as the use of an incorrect substitution model (or the model's assumptions) [17] or improper priors could lead to incorrect inferences [18].

A character, or mutational, map represents the character's history across the phylogeny. What we would like to accomplish is to sample character histories in proportion to their posterior probabilities. Formally, we would like to evaluate the following distribution,

Pr(M|D)=∑k∈ψ∫vk∫θPr(M|D,θ,τ,)p(τk,vk,θ|D)dvkdθ,     (1)
 MathType@MTEF@5@5@+=feaafiart1ev1aaatCvAUfKttLearuWrP9MDH5MBPbIqV92AaeXatLxBI9gBaebbnrfifHhDYfgasaacH8akY=wiFfYdH8Gipec8Eeeu0xXdbba9frFj0=OqFfea0dXdd9vqai=hGuQ8kuc9pgc9s8qqaq=dirpe0xb9q8qiLsFr0=vr0=vr0dc8meaabaqaciaacaGaaeqabaqabeGadaaakeaatCvAUfeBSjuyZL2yd9gzLbvyNv2CaeHbwvMCKfMBHbaceiGaa8huaiaa=jhacqGGOaakcqWGnbqtcqGG8baFcqWGebarcqGGPaqkcqGH9aqpdaaeqbqaamaapebabaWaa8qeaeaacaWFqbGaa8NCaiabcIcaOiabd2eanjabcYha8jabdseaejabcYcaSGGaciab+H7aXjabcYcaSiab+r8a0bWcbaGae4hUdehabeqdcqGHRiI8aaWcbaGaemODayNaem4AaSgabeqdcqGHRiI8aOGaeiilaWcaleaacqWGRbWAcqGHiiIZcqGFipqEaeqaniabggHiLdGccqGGPaqkcqWGWbaCcqGGOaakcqGFepaDdaWgaaWcbaGaem4AaSgabeaakiabcYcaSiabdAha2naaBaaaleaacqWGRbWAaeqaaOGaeiilaWIae4hUdeNaeiiFaWNaemiraqKaeiykaKIaemizaqMaemODay3aaSbaaSqaaiabdUgaRbqabaGccqWGKbazcqGF4oqCcqGGSaalcaWLjaGaaCzcamaabmaabaGaeGymaedacaGLOaGaayzkaaaaaa@779B@

where *M *are character histories, *D *is the data, *θ *is a vector containing parameters of the substitution model, *τ *is the topology, *v *is the vector of branch lengths associated with *τ*, and *ψ *is the set of possible topologies. The dimensionality of the problem, for all but the simplest topologies, prohibits a direct analytical evaluation. Fortunately, methods such as MCMC can be used to sample topologies, branch lengths, model parameters, and character histories [15]. The following describes the algorithm implemented in SIMMAP.

Given a topology, branch lengths, and substitution model parameters a character history can be sampled using the following three-step algorithm. Briefly,

1. Calculate conditional likelihood for each character state at each node of the tree, including the root,

2. Simulate ancestral states at each internal node by sampling from the posterior distribution of states, and

3. Simulate a substitution (mutational) history, by sampling from the posterior distribution conditioned on the reconstructions in step 2 and observed states at the tips of the topology. The waiting times between substitutions are drawn from an exponential distribution with the rate being the diagonal elements of the model's instantaneous rate matrix (**Q**), conditioned on the current state (see below).

Often the states at the tips of the tree are unknown or uncertain (e.g., ?, N, R, etc). This type of uncertainty can easily be accommodated by revisiting these nodes after simulating ancestral states for the internal nodes and repeating Step 2 for the tips. By following this algorithm we have obtained a single sample (or realization) of a character history.

Let us look more closely at each of these steps. The first step in the algorithm requires the calculation of conditional likelihoods, *l*, of each state at each internal node of the tree. Given instantaneous rates of substitution given by the model's **Q **matrix and the length of the branch (*v*) associated with the node we can easily calculate substitution probabilities as **P **= *p*_*ij*_(*v*) = *e*^**Q***v*^. In a number of cases, such as the JC69 model [19], analytical solutions have been described. In cases in which analytical solutions are unavailable standard linear algebra approaches for exponentiating matrices can be used (e.g., LAPACK [20]). With these probabilities we can efficiently calculate the conditional likelihoods using the post-order traversal *pruning algorithm *of Felsenstein [21].

Next, we want to sample ancestral states at the internal nodes of the tree using the conditional likelihoods calculated in step 1. Starting at the root of the tree, denoted as *σ*, we stochastically simulate a state *d *using the following,

Pr(dσ=i|D,τ,θ)=lσ,iπi∑j∈Ωlσ,jπj,     (2)
 MathType@MTEF@5@5@+=feaafiart1ev1aaatCvAUfKttLearuWrP9MDH5MBPbIqV92AaeXatLxBI9gBaebbnrfifHhDYfgasaacH8akY=wiFfYdH8Gipec8Eeeu0xXdbba9frFj0=OqFfea0dXdd9vqai=hGuQ8kuc9pgc9s8qqaq=dirpe0xb9q8qiLsFr0=vr0=vr0dc8meaabaqaciaacaGaaeqabaqabeGadaaakeaatCvAUfeBSjuyZL2yd9gzLbvyNv2CaeHbwvMCKfMBHbaceiGaa8huaiaa=jhacqGGOaakcqWGKbazdaWgaaWcbaacciGae43Wdmhabeaakiabg2da9iabdMgaPjabcYha8jabdseaejabcYcaSiab+r8a0jabcYcaSiab+H7aXjabcMcaPiabg2da9maalaaabaGaemiBaW2aaSbaaSqaaiab+n8aZjabcYcaSiabdMgaPbqabaGccqGFapaCdaWgaaWcbaGaemyAaKgabeaaaOqaamaaqababaGaemiBaW2aaSbaaSqaaiab+n8aZjabcYcaSiabdQgaQbqabaGccqGFapaCdaWgaaWcbaGaemOAaOgabeaaaeaacqWGQbGAcqGFiiIZcqGHPoWvaeqaniabggHiLdaaaOGaeiilaWIaaCzcaiaaxMaadaqadaqaaiabikdaYaGaayjkaiaawMcaaaaa@65C7@

where, for example, *Ω *= {*A, C, G, T*} for DNA.

Once we have sampled a state at the root, *d*_*σ *_we simulate ancestral states at each internal node using a pre-order traversal tree by sampling from the following,

Pr(dσ−1=j|dσ=i,D,τ,θ)=lσ−1,iPij(tσ−1)∑k∈Ωlσ−1,kPik(tσ−1).     (3)
 MathType@MTEF@5@5@+=feaafiart1ev1aaatCvAUfKttLearuWrP9MDH5MBPbIqV92AaeXatLxBI9gBaebbnrfifHhDYfgasaacH8akY=wiFfYdH8Gipec8Eeeu0xXdbba9frFj0=OqFfea0dXdd9vqai=hGuQ8kuc9pgc9s8qqaq=dirpe0xb9q8qiLsFr0=vr0=vr0dc8meaabaqaciaacaGaaeqabaqabeGadaaakeaatCvAUfeBSjuyZL2yd9gzLbvyNv2CaeHbwvMCKfMBHbaceiGaa8huaiaa=jhacqGGOaakcqWGKbazdaWgaaWcbaacciGae43WdmNae4NeI0IaeGymaedabeaakiabg2da9iabdQgaQjabcYha8jabdsgaKnaaBaaaleaacqGFdpWCaeqaaOGaeyypa0JaemyAaKMaeiilaWIaemiraqKaeiilaWIae4hXdqNaeiilaWIae4hUdeNaeiykaKIaeyypa0ZaaSaaaeaacqWGSbaBdaWgaaWcbaGae43WdmNaeyOeI0IaeGymaeJaeiilaWIaemyAaKgabeaakiabdcfaqnaaBaaaleaacqWGPbqAcqWGQbGAaeqaaOGaeiikaGIaemiDaq3aaSbaaSqaaiab+n8aZjabgkHiTiabigdaXaqabaGccqGGPaqkaeaadaaeqaqaaiabdYgaSnaaBaaaleaacqGFdpWCcqGFsislcqaIXaqmcqGGSaalcqWGRbWAaeqaaOGaemiuaa1aaSbaaSqaaiabdMgaPjabdUgaRbqabaGccqGGOaakcqWG0baDdaWgaaWcbaGae43WdmNae4NeI0IaeGymaedabeaakiabcMcaPaWcbaGaem4AaSMae4hcI4SaeyyQdCfabeqdcqGHris5aaaakiabc6caUiaaxMaacaWLjaWaaeWaaeaacqaIZaWmaiaawIcacaGLPaaaaaa@8170@

Now, we have assigned ancestral states at the internal nodes. The final step of the algorithm we will simulate a realization of character history along each branch (using a pre-order traversal) conditional on the ancestral reconstructions and observations at the tips of the topology. Each realization is simulated using a continuous-time Markov chain in which substitutions are modeled as a Poisson process. The waiting times until a character change along a branch for the Poisson is

*λe*^*λv *^    (4)

with the rate *λ *= *-q*_*ii*_. This rate is derived from the diagonal elements of the model's **Q **matrix. These elements can be interpreted as the overall rate of changing from state *i*. Waiting times are simulated using the inverse transformation method. If the waiting time is longer than the branch length, *v*, and the states at each end of the branch are the same, then the realization is complete; no changes have occurred along this branch. However, if the waiting time is smaller than the branch length, *v*, then a substitution is drawn from, pij=qij−qii
 MathType@MTEF@5@5@+=feaafiart1ev1aaatCvAUfKttLearuWrP9MDH5MBPbIqV92AaeXatLxBI9gBaebbnrfifHhDYfgasaacH8akY=wiFfYdH8Gipec8Eeeu0xXdbba9frFj0=OqFfea0dXdd9vqai=hGuQ8kuc9pgc9s8qqaq=dirpe0xb9q8qiLsFr0=vr0=vr0dc8meaabaqaciaacaGaaeqabaqabeGadaaakeaacqWGWbaCdaWgaaWcbaGaemyAaKMaemOAaOgabeaakiabg2da9maalaaabaGaemyCae3aaSbaaSqaaiabdMgaPjabdQgaQbqabaaakeaacqGHsislcqWGXbqCdaWgaaWcbaGaemyAaKMaemyAaKgabeaaaaaaaa@3BAC@, and a new waiting time is simulated using the new length, *v - v*_1_. If this next waiting time exceeds the remaining length of the branch, and the states are the same, the realization is complete. On the other hand, if the states are different, the process is repeated from the ancestral node, because the realization is not consistent with the state at the descendant node. If we were to proceed from the previous transformation on the branch the waiting times would no longer be exponentially distributed.

While we could sample a character history without first sampling ancestral states (Step 2 above) this would substantially increase the computational time required. The reason for this is that most of the histories would be rejected because they are not consistent with the observed data. This would require a new history for the whole tree to be re-sampled rather than simply rejecting the history along a single branch as in the algorithm above. It should be remembered that we want to sample histories conditional on the observations at the tips of the tree – *Pr*(*History*|*Data*). By sampling ancestral states from their posterior distribution first we are reducing the number of histories that are rejected and increasing the acceptance of samples from the desired probability distribution.

Often, we are primarily interested in inferring some aspect of a character's evolution and not the particulars of the phylogeny or other parameters. We would like to avoid making inferences based on a single topology because it is rarely known with certainty. Rather we would like to integrate over this uncertainty. Therefore, to accommodate uncertainty in both model parameters and the phylogeny SIMMAP samples character histories for each parameter sample from the posterior distribution; topologies and parameter values are thus sampled in proportion to their posterior probabilities. Samples from these distributions can be obtained by MCMC using software such as MrBayes [22]. A sample of character histories obtained in this way can then be summarized to test a variety of hypotheses, such as, the number of gains and losses of a trait, correlated character evolution, number of synonymous and non-synonymous changes, patterns of amino acid evolution (e.g., rates of conservative versus radical substitution), and the placement and order of changes on the phylogeny. In this latter type of analysis we are interested in only those histories that are consistent with presence of a particular group. The absence of a group in a particular topology sampled from the posterior poses no problem as the hypothesis is now conditional on its presence; topologies not consistent with the presence of a group can be ignored and the resulting sampled histories are from the conditional distribution.

In addition to implementing the above approach of stochastic posterior character mapping, SIMMAP also allows sampling from posterior predictive densities to evaluate the significance of hypotheses [16,23]. Briefly, this method generates a distribution of character histories under a null hypothesis which are summarized by a test statistic and then compared to the observed test statistic. For example, it is straightforward to test for nonrandom associations between characters (see below). The predictive probability of the observed test statistic can be calculated using the tail areas of the statistic under the null model. (For a more detailed description of predictive distributions, see [24].)

## Implementation

### Brief overview

SIMMAP is Graphical User Interface (GUI) program written to run on the Apple's OS X operating system. SIMMAP reads in 1) a NEXUS data file containing a matrix of DNA, RNA, or morphological (standard) characters, 2) a file containing trees in the Newick format, and 3) a text file containing DNA substitution model parameter values. Data, trees, and parameters are displayed graphically and can be manipulated by the user. For example, characters and taxa can be included, excluded, and trees can be rooted and a variety of, *a priori*, branch lengths can be applied. Trees and character maps are drawn with user customized labels at the tips, font sizes, branch length thickness, and color schemes for easy identification of character states.

SIMMAP can be used to sample character histories on phylogenies to address questions about evolutionary patterns of change, positive selection at focal sites or across a gene region, and correlation among characters. In addition, SIMMAP can be used to calculate the posterior distribution of ancestral states. SIMMAP is a software implementation of the approach described by Nielsen [23] and Huelsenbeck et al. [16] with a couple of minor extensions. First, SIMMAP effectively deals with uncertain or unknown character states at the tips of the tree (see above). Second, new test statistics for measuring covariation between characters are implemented as described below.

### Substitution models

SIMMAP implements stochastic substitution models for both molecular and standard characters. When analyzing nucleotide data the user can implement the most general 4 × 4 model of DNA sequence evolution [25], and all sub-models. Among site rate variation can be accommodated using a discrete gamma [26], invariable sites, or site-specific models [27]. Parameters for the models can be set manually by the user, read in from the contents of a file, or a mixture of the two.

Morphological character evolution is implemented using the *M*_*k *_class of models [28], with up to 7 states. Two priors are placed on morphological models: a discrete gamma prior on the overall rate of morphological character evolution, and a discrete symmetrical beta prior on the bias parameter of the *M*_2_model [16,29]. The user can customize these priors by selecting the parameters of the prior distributions and the number of categories used to make the distribution discrete. The number of samples drawn from the priors can be adjusted to allow a sufficient sampling approximation of the posterior distribution on bias and rates. For binary characters, the posterior distribution of the bias parameter can be used to make inferences about the pattern of evolution while accommodating uncertainty in the underlying phylogeny and branch lengths.

There is no clear rule of thumb as to the values of the parameters for the prior distributions or how many samples should be drawn to provide a sufficient approximation. Users should try a variety of parameter values and sample sizes to ensure that their inferences are not changing dramatically.

### Character histories

SIMMAP allows the user to explore single character histories visually one at time, or perform multiple realizations of sampling from the posterior of character histories. The analyses can be tailored to evaluate a single character (nucleotide, codon, or standard character) or multiple characters, on a single tree or a set of trees, over a set of model parameters or priors, and with a user defined number of samples.

SIMMAP summarizes character histories across the phylogeny using the posterior expectation of different statistics (Table 1). Briefly, SIMMAP reports the expected number of changes (e.g., Ile ? Leu), the direction of changes if the tree is rooted (e.g., Ile ? Leu), the types and expected number of changes on each branch (e.g., number of non-synonymous/synonymous changes), and the correlation between characters. In addition to these summary statistics the details of each individual history can be written to a file as a tab delimited file for analysis by an external program. SIMMAP also offers the option of saving the raw character history data in a modified Newick tree format. For example, a character history for a three taxon tree is represented in the following way:

**Table 1:** SIMMAP summary statistics

Data Type	Statistic	Output*
Nucleotide	E [*i *→ *j*]	1,2
	E[*transition*]	1,2
	E[*transversion*]	1,2
	E[*time*]	1,2
	E[*subs*/*branch*]	1

Codon	E[*i → j*]	1,2
	E[*synonymous*]	1,2
	E[*non-synonymous*]	1,2
	E[*time*]	1,2
	E[*conservative*]	1,2
	E[*radical*]	1,2
	E[*AA*_*i *_→ *AA*_*j*_]	1,2
	E[*Δhydropathy*]	1,2

Standard (Morphology)	E [*i → j*]	1,2
	E[*time*]	1,2
	E[*bias*]	1,2
	E[*rate*]	1,2

* Statistics can be displayed on screen (1) or saved to a file (2). Values reported are the posterior expectations.

((*Taxon1*:{A,0.1;C,0.1}, *Taxon2*:{T,0.1;C,0.1}):{C,0.1}, *Taxon3:*{C,0.4})

where a branch is divided into intervals representing the character's history along the branch. The history is described in the curly brackets following a tip or internal node of the tree in a tip to root encoding. For example, from the tip node, *Taxon2*, backward in time to its ancestor the mutational history along this branch is described as {T,0.1;C,0.1}; from the tip to the ancestor a T is observed for a length of 0.1 followed by a C with a length of 0.1 terminating in this state.

Significance is assigned using predictive *p*-values which can be selected to test hypotheses about the number of synonymous/non-synonymous changes, nucleotide character association (correlation), and morphological character association. The user can customize the design of the predictive test by controlling the number of replicates, which trees, model parameters, and prior values are used.

SIMMAP provides the user the ability to place constraints on a number of aspects of the analysis; the user can 1) fix ancestral states at individual nodes of a tree to a particular state or set of states, 2) constrain the analysis to particular trees by defining clade constraints, and 3) root trees using one or more outgroup species. In addition, SIMMAP will calculate the posterior probability of ancestral states using an empirical or hierarchical Bayesian approach.

### Character correlation statistics

Often we would like to know whether two characters, or particular character states, covary on the phylogeny. For example, morphological studies addressing hypotheses regarding correlated evolution or ecological studies testing for associations between a character and a particular environment. Character histories can be used to address these questions using SIMMAP. A number of measures of correlation are implemented for nucleotide and morphological characters and their states.

Statistics for measuring morphological character and state associations, first described by Huelsenbeck *et al*. [16], have been implemented in SIMMAP to test for state-by-state associations (eqn. 5) and overall character correlation (eqn. 6):

dij=aij(o)−aij(e),     (5)
 MathType@MTEF@5@5@+=feaafiart1ev1aaatCvAUfKttLearuWrP9MDH5MBPbIqV92AaeXatLxBI9gBaebbnrfifHhDYfgasaacH8akY=wiFfYdH8Gipec8Eeeu0xXdbba9frFj0=OqFfea0dXdd9vqai=hGuQ8kuc9pgc9s8qqaq=dirpe0xb9q8qiLsFr0=vr0=vr0dc8meaabaqaciaacaGaaeqabaqabeGadaaakeaacqWGKbazdaWgaaWcbaGaemyAaKMaemOAaOgabeaakiabg2da9iabdggaHnaaDaaaleaacqWGPbqAcqWGQbGAaeaacqGGOaakcqWGVbWBcqGGPaqkaaGccqGHsislcqWGHbqydaqhaaWcbaGaemyAaKMaemOAaOgabaGaeiikaGIaemyzauMaeiykaKcaaOGaeiilaWIaaCzcaiaaxMaadaqadaqaaiabiwda1aGaayjkaiaawMcaaaaa@4615@

D=∑i=1n∑j=1m|dij|,     (6)
 MathType@MTEF@5@5@+=feaafiart1ev1aaatCvAUfKttLearuWrP9MDH5MBPbIqV92AaeXatLxBI9gBaebbnrfifHhDYfgasaacH8akY=wiFfYdH8Gipec8Eeeu0xXdbba9frFj0=OqFfea0dXdd9vqai=hGuQ8kuc9pgc9s8qqaq=dirpe0xb9q8qiLsFr0=vr0=vr0dc8meaabaqaciaacaGaaeqabaqabeGadaaakeaacqWGebarcqGH9aqpdaaeWbqaamaaqahabaGaeiiFaWNaemizaq2aaSbaaSqaaiabdMgaPjabdQgaQbqabaaabaGaemOAaOMaeyypa0JaeGymaedabaGaemyBa0ganiabggHiLdGccqGG8baFcqGGSaalcaWLjaGaaCzcamaabmaabaGaeGOnaydacaGLOaGaayzkaaaaleaacqWGPbqAcqGH9aqpcqaIXaqmaeaacqWGUbGBa0GaeyyeIuoaaaa@488E@

where, aij(o)
 MathType@MTEF@5@5@+=feaafiart1ev1aaatCvAUfKttLearuWrP9MDH5MBPbIqV92AaeXatLxBI9gBaebbnrfifHhDYfgasaacH8akY=wiFfYdH8Gipec8Eeeu0xXdbba9frFj0=OqFfea0dXdd9vqai=hGuQ8kuc9pgc9s8qqaq=dirpe0xb9q8qiLsFr0=vr0=vr0dc8meaabaqaciaacaGaaeqabaqabeGadaaakeaacqWGHbqydaqhaaWcbaGaemyAaKMaemOAaOgabaGaeiikaGIaem4Ba8MaeiykaKcaaaaa@33F5@ is the observed association between character state *i *and *j*, aij(e)
 MathType@MTEF@5@5@+=feaafiart1ev1aaatCvAUfKttLearuWrP9MDH5MBPbIqV92AaeXatLxBI9gBaebbnrfifHhDYfgasaacH8akY=wiFfYdH8Gipec8Eeeu0xXdbba9frFj0=OqFfea0dXdd9vqai=hGuQ8kuc9pgc9s8qqaq=dirpe0xb9q8qiLsFr0=vr0=vr0dc8meaabaqaciaacaGaaeqabaqabeGadaaakeaacqWGHbqydaqhaaWcbaGaemyAaKMaemOAaOgabaGaeiikaGIaemyzauMaeiykaKcaaaaa@33E1@ is the expected association, *n *is the number of states for character 1 and *m *is the number of states for character 2. The association between one state and another is the frequency of occurrence of state *i *and state *j *on the phylogeny.

When analyzing nucleotide data the following statistics have been implemented for the overall correlation (7) and state-by-state correlations (8):

xij=(aij(o)−aij(e))2(aij(e)+C),     (7)
 MathType@MTEF@5@5@+=feaafiart1ev1aaatCvAUfKttLearuWrP9MDH5MBPbIqV92AaeXatLxBI9gBaebbnrfifHhDYfgasaacH8akY=wiFfYdH8Gipec8Eeeu0xXdbba9frFj0=OqFfea0dXdd9vqai=hGuQ8kuc9pgc9s8qqaq=dirpe0xb9q8qiLsFr0=vr0=vr0dc8meaabaqaciaacaGaaeqabaqabeGadaaakeaacqWG4baEdaWgaaWcbaGaemyAaKMaemOAaOgabeaakiabg2da9maalaaabaGaeiikaGIaemyyae2aa0baaSqaaiabdMgaPjabdQgaQbqaaiabcIcaOiabd+gaVjabcMcaPaaakiabgkHiTiabdggaHnaaDaaaleaacqWGPbqAcqWGQbGAaeaacqGGOaakcqWGLbqzcqGGPaqkaaGccqGGPaqkdaahaaWcbeqaaiabikdaYaaaaOqaaiabcIcaOiabdggaHnaaDaaaleaacqWGPbqAcqWGQbGAaeaacqGGOaakcqWGLbqzcqGGPaqkaaGccqGHRaWkcqWGdbWqcqGGPaqkaaGaeiilaWIaaCzcaiaaxMaadaqadaqaaiabiEda3aGaayjkaiaawMcaaaaa@540E@

χ2=∑i=1n∑j=1mxij,     (8)
 MathType@MTEF@5@5@+=feaafiart1ev1aaatCvAUfKttLearuWrP9MDH5MBPbIqV92AaeXatLxBI9gBaebbnrfifHhDYfgasaacH8akY=wiFfYdH8Gipec8Eeeu0xXdbba9frFj0=OqFfea0dXdd9vqai=hGuQ8kuc9pgc9s8qqaq=dirpe0xb9q8qiLsFr0=vr0=vr0dc8meaabaqaciaacaGaaeqabaqabeGadaaakeaaiiGacqWFhpWydaahaaWcbeqaaiabikdaYaaakiabg2da9maaqahabaWaaabCaeaacqWG4baEdaWgaaWcbaGaemyAaKMaemOAaOgabeaaaeaacqWGQbGAcqGH9aqpcqaIXaqmaeaacqWGTbqBa0GaeyyeIuoaaSqaaiabdMgaPjabg2da9iabigdaXaqaaiabd6gaUbqdcqGHris5aOGaeiilaWIaaCzcaiaaxMaadaqadaqaaiabiIda4aGaayjkaiaawMcaaaaa@4790@

where, *C*, is a positive constant representing Yate's correction term for continuity (C = 0.5).

In addition, SIMMAP implements a statistic similar in form to the mutual information content statistic (MIC) for both nucleotide and morphological data types. As implemented in SIMMAP, this statistic evaluates the correlation between character histories along the phylogeny for two characters. This statistic takes the following form for state-by-state correlations,

mij=fijlog⁡2fijfifj,     (9)
 MathType@MTEF@5@5@+=feaafiart1ev1aaatCvAUfKttLearuWrP9MDH5MBPbIqV92AaeXatLxBI9gBaebbnrfifHhDYfgasaacH8akY=wiFfYdH8Gipec8Eeeu0xXdbba9frFj0=OqFfea0dXdd9vqai=hGuQ8kuc9pgc9s8qqaq=dirpe0xb9q8qiLsFr0=vr0=vr0dc8meaabaqaciaacaGaaeqabaqabeGadaaakeaacqWGTbqBdaWgaaWcbaGaemyAaKMaemOAaOgabeaakiabg2da9iabdAgaMnaaBaaaleaacqWGPbqAcqWGQbGAaeqaaOGagiiBaWMaei4Ba8Maei4zaC2aaSbaaSqaaiabikdaYaqabaGcdaWcaaqaaiabdAgaMnaaBaaaleaacqWGPbqAcqWGQbGAaeqaaaGcbaGaemOzay2aaSbaaSqaaiabdMgaPbqabaGccqWGMbGzdaWgaaWcbaGaemOAaOgabeaaaaGccqGGSaalcaWLjaGaaCzcamaabmaabaGaeGyoaKdacaGLOaGaayzkaaaaaa@4A5A@

where *f*_*ij *_is the fraction of time state *i *is associated with *j *in a character history, and *f*_*i *_(*f*_*j*_) is the fraction time in a particular state independent of associations. By averaging over all state associations we get the following statistic for the overall character correlation:

M=∑i=1x∑j=1ymij.     (10)
 MathType@MTEF@5@5@+=feaafiart1ev1aaatCvAUfKttLearuWrP9MDH5MBPbIqV92AaeXatLxBI9gBaebbnrfifHhDYfgasaacH8akY=wiFfYdH8Gipec8Eeeu0xXdbba9frFj0=OqFfea0dXdd9vqai=hGuQ8kuc9pgc9s8qqaq=dirpe0xb9q8qiLsFr0=vr0=vr0dc8meaabaqaciaacaGaaeqabaqabeGadaaakeaacqWGnbqtcqGH9aqpdaaeWbqaamaaqahabaGaemyBa02aaSbaaSqaaiabdMgaPjabdQgaQbqabaaabaGaemOAaOMaeyypa0JaeGymaedabaGaemyEaKhaniabggHiLdaaleaacqWGPbqAcqGH9aqpcqaIXaqmaeaacqWG4baEa0GaeyyeIuoakiabc6caUiaaxMaacaWLjaWaaeWaaeaacqaIXaqmcqaIWaamaiaawIcacaGLPaaaaaa@46C6@

Regardless of the statistic used significance is determined by sampling from the posterior predictive distribution of the statistic to form a null distribution of no character (or state) association. For example, predictive *p*-values for the *M *statistic would be calculated in the following way,

p=1N∑i=1NI(T(Mobs)≥T(Mi)),     (11)
 MathType@MTEF@5@5@+=feaafiart1ev1aaatCvAUfKttLearuWrP9MDH5MBPbIqV92AaeXatLxBI9gBaebbnrfifHhDYfgasaacH8akY=wiFfYdH8Gipec8Eeeu0xXdbba9frFj0=OqFfea0dXdd9vqai=hGuQ8kuc9pgc9s8qqaq=dirpe0xb9q8qiLsFr0=vr0=vr0dc8meaabaqaciaacaGaaeqabaqabeGadaaakeaacqWGWbaCcqGH9aqpdaWcaaqaaiabigdaXaqaaiabd6eaobaadaaeWbqaaiabdMeajjabcIcaOiabdsfaujabcIcaOiabd2eannaaBaaaleaacqWGVbWBcqWGIbGycqWGZbWCaeqaaOGaeiykaKIaeyyzImRaemivaqLaeiikaGIaemyta00aaSbaaSqaaiabdMgaPbqabaaabaGaemyAaKMaeyypa0JaeGymaedabaGaemOta4eaniabggHiLdGccqGGPaqkcqGGPaqkcqGGSaalcaWLjaGaaCzcamaabmaabaGaeGymaeJaeGymaedacaGLOaGaayzkaaaaaa@5003@

where *N *is the number of realizations. In practice, the expectations of the statistics are used to summarize histories over trees, model parameters, and, if applicable, priors.

### Documentation and help

Finally, a detailed Help Manual is provide in three formats: integrated with the program through Apple help, HTML documentation included with the electronic distribution, or on the web [30]. Users can browse help topics by aspects of the interface (e.g., windows) or follow a brief tutorial to give users a quick start to performing mapping analysis with SIMMAP. In addition, a section of the documentation provides a brief description of the methodology of sampling stochastic character histories and a list of other relevant publications.

## Conclusion

SIMMAP provides a needed tool for mapping character histories on phylogenies that is not dependent on the parsimony method. Using a Bayesian approach, the application described here enables researchers to address a wide variety of questions important in evolutionary studies. In addition, SIMMAP can be used as a teaching tool for explaining stochastic models, Bayesian inference, and character histories.

## Availability and requirements

**Program name: **SIMMAP

**Current Version: **1.0 Beta 2.0.4

**Program home page: **

**Operating System: **Apple Macintosh OS X 10.3 or greater

**Programming language: **C, Objective-C, Cocoa Frameworks

**License: **SIMMAP Free Academic End-user License

**Any restrictions to use by non-academics: **License required. Consult the documentation in the software distribution.

## List of abbreviations

MCMC: Markov chain Monte Carlo

**Q **matrix: A rate matrix describing the instantaneous rates of changing from one state to another for a continuous-time Markov model.

JC69: Jukes-Cantor substitution model described in 1969 [19]. Assumes equal probabilities of change between states.
